# The stressed brain of humans and rodents

**DOI:** 10.1111/apha.13066

**Published:** 2018-04-16

**Authors:** M. Joëls, H. Karst, R. A. Sarabdjitsingh

**Affiliations:** ^1^ Department of Translational Neuroscience Brain Center Rudolf Magnus University Medical Center Utrecht Utrecht University Utrecht The Netherlands; ^2^ University Medical Center Groningen University of Groningen Groningen The Netherlands

**Keywords:** amygdala, corticosterone, cortisol, early life stress, hippocampus

## Abstract

After stress, the brain is exposed to waves of stress mediators, including corticosterone (in rodents) and cortisol (in humans). Corticosteroid hormones affect neuronal physiology in two time‐domains: rapid, non‐genomic actions primarily via mineralocorticoid receptors; and delayed genomic effects via glucocorticoid receptors. In parallel, cognitive processing is affected by stress hormones. Directly after stress, emotional behaviour involving the amygdala is strongly facilitated with cognitively a strong emphasis on the “now” and “self,” at the cost of higher cognitive processing. This enables the organism to quickly and adequately respond to the situation at hand. Several hours later, emotional circuits are dampened while functions related to the prefrontal cortex and hippocampus are promoted. This allows the individual to rationalize the stressful event and place it in the right context, which is beneficial in the long run. The brain's response to stress depends on an individual's genetic background in interaction with life events. Studies in rodents point to the possibility to prevent or reverse long‐term consequences of early life adversity on cognitive processing, by normalizing the balance between the two receptor types for corticosteroid hormones at a critical moment just before the onset of puberty.

## 1 INTRODUCTION

Humans and rodents are continuously exposed to changes in their environment, introducing potential threats—real or perceived—to bodily processes. These threats, stressors, are subjectively experienced as “stress.” The response to stress is quite conserved among mammals.^1^, ^2^ Information about the threatening situation is funnelled through the hypothalamus and from there first leads to activation of the sympathetic nervous system, causing the quick release of adrenaline from the adrenal medulla; slightly later, the hypothalamus‐pituitary‐adrenal system is activated, resulting in synthesis and release of steroid hormones from the adrenal cortex. Corticosterone is the main corticosteroid hormone in rats and mice, while cortisol is the predominant adrenal stress hormone in humans. The waves of adrenaline (noradrenaline in the brain) and corticosteroids not only reach peripheral organs but also reach cells in the brain. Consequently, cells carrying receptors for these transmitters and hormones are expected to change in function after stress, which eventually will have impact on behaviour. The behavioural response enables the individual to adapt to a changing environment.

Much has become known over the past decades about the (two) types of corticosteroid receptors in the brain.^3^ The mineralocorticoid receptor (MR) has a restricted distribution, with high expression levels in, for example the hippocampus, lateral septum and lower levels in cortical layers and the amygdala. The glucocorticoid receptor (GR) is more ubiquitously expressed, with particularly high expression in the paraventricular nucleus (PVN) of the hypothalamus and some regions of the hippocampus. MRs have a very high affinity for corticosterone and cortisol as well as the less prevalent adrenal steroid aldosterone. Due to its high affinity, this receptor type is already substantially occupied by corticosterone (rodents) or cortisol (humans) even in non‐stressed individuals. By contrast, the GR has a 10‐fold lower affinity, so that under non‐stressed conditions, this receptor type is only partially occupied but becomes fully activated after stress. Both MR and GR, when bound to corticosteroid hormones, move to the nucleus where they act as transcriptional regulators, changing the expression of large networks of genes at a time. On top of (i) the regional distribution of MR and GR in brain, (ii) their affinity and (iii) the release pattern of corticosteroids from the adrenal glands, there are many other factors that eventually shape the brain's response to corticosteroids after stress, including (iv) the circulating levels of corticosteroid‐binding protein in blood, (v) the presence of p‐glycoproteins in epithelial cells determining the accessibility of corticosteroids to the brain and neurons, (vi) corticosteroid‐converting enzymes such as 11‐β‐hydroxysteroid dehydrogenase and (vii) transcriptional coregulators involved in the process downstream of receptor activation.^2^, ^4^, ^5^ All of these factors together determine how individual neurons will respond to shifts in corticosteroid level after stress.

## 2 LESSONS LEARNED FROM CELLULAR PHYSIOLOGY

Since the late 1970s, many electrophysiological studies have been performed to delineate exactly how corticosteroid hormones affect neuronal activity. A comprehensive overview is provided elsewhere.^2^ Here, we will only highlight some leading principles that emerged from this body of work.

**Figure 1:**
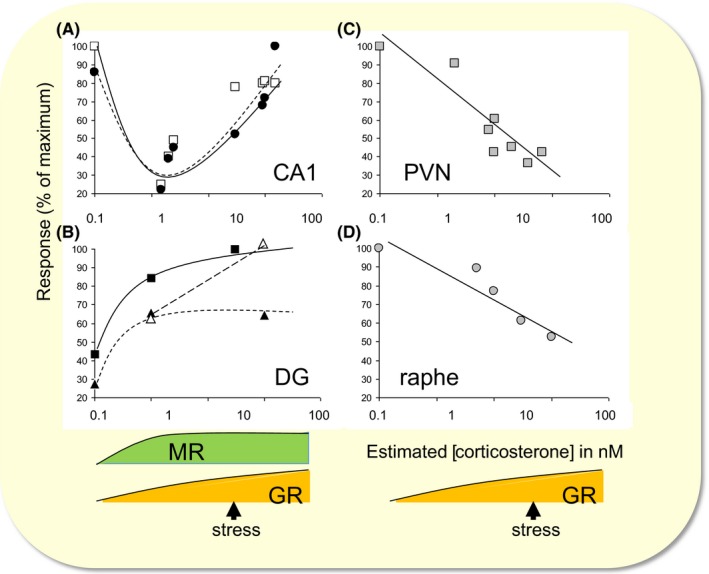
Dose‐response relationships of cellular effects by corticosterone in the brain. Dose‐response relationships are shown for A, the CA1 hippocampal area, B, the dentate gyrus (DG), C, the paraventricular nucleus (PVN) of the hypothalamus and D, the dorsal raphe nucleus. Graphs show hormone responses expressed as a percentage of the maximal response in these brain regions. The concentration of corticosterone is an approximate estimate of the local concentration based on the solutions perfused on in vitro preparations or derived from the plasma concentration when fluctuations in hormone levels were accomplished in vivo. A, In the CA1 area, both the amplitude of depolarization‐induced calcium currents (white squares) and the hyperpolarization caused by serotonin‐1A receptor activation (black circles) display a U‐shaped dose dependency. The descending limb is linked to the activation of mineralocorticoid receptors (MRs), whereas the ascending limb is associated with gradual glucocorticoid receptor (GR) activation in addition to already activated MRs, as occurs after stress. B, DG granule neurons show a clear MR‐dependent effect on the field potential (black squares) and the single‐cell response (black triangles) caused by activation of glutamate AMPA receptors. Although these cells also abundantly express GRs, high doses of corticosterone do not cause additional changes in the signal, except when tested in chronically stressed rats (white triangles). A similar phenomenon was found for calcium currents (see main text). C, Neurons in the PVN and D, the raphe nucleus express GRs primarily. In these cells, a linear dose dependency is seen for the frequency of spontaneous γ‐aminobutyric acid (GABA)A‐receptor‐mediated synaptic events (grey squares)and the inhibition caused by serotonin‐1A receptor activation (grey circles). Adapted and reproduced with permission from reference 6

The first principle is that neurons respond to corticosteroids in a concentration‐dependent manner. The dose dependency, however, is regionally differentiated (Figure 1).^6^ To some extent, this is linked to the affinity range of the two receptors types and their difference in distribution pattern across the brain. For instance, the response of neurons in the raphe nucleus to serotonin is affected by corticosteroids in a linear fashion, probably linked to the degree of activation of the GR which prevails in these cells. By contrast, CA1 hippocampal principal cells abundantly express both MR and GR. In these cells too, the response to serotonin—or other properties, such as the amplitude of voltage‐gated calcium currents—is more or less linearly affected by a shift in the concentration range from the level seen at non‐stressed to stressed conditions. However, when concentrations drop below the non‐stressed level, serotonin responses and voltage‐gated calcium currents do not diminish in a linear fashion but become bigger due to inactivation of the MR, overall resulting in a U‐shaped dose dependency. These conditions where corticosteroid concentrations are so low that not even MRs are activated probably rarely occur under physiological conditions but can be revealed when hormone levels are drastically reduced by adrenalectomy. The dose dependency is not only governed by the distribution pattern of the receptors, however; other region‐dependent factors also play a role in the overall response to the hormone. For example, granule cells in the dentate gyrus (similar to CA1 pyramidal cells) also express high levels of both MR and GR. The shift in responses between conditions where the MR is unoccupied to the situation with low circulating corticosteroid levels so that MRs are substantially activated while GRs are still mostly inactivated is comparable to the CA1 area. However, when corticosteroid levels rise even further and activate the remainder of the GR pool, no change in the cellular properties was observed. This was studied in more detail for the effect of corticosteroids on voltage‐gated L‐type calcium currents.^7^ It was observed that up to the transcriptional level, the response of granule cells was highly comparable to that of CA1 neurons. However, the step from transcription to translation was apparently hampered, as in the dentate gyrus, no effect of GR activation was apparent at the protein or functional level.

A second principle relates to the time‐domain over which corticosteroid hormones change cellular function. Most cellular actions described develop with a delay of approx. 1 hours which is compatible with the gene‐mediated signalling pathway. However, even some of the earlier reports showed that corticosteroids can also rapidly modify neuronal firing. Studies from Jeffrey Tasker and colleagues revealed that in parvocellular neurons of the PVN, corticosterone or dexamethasone decreased the release probability of glutamate‐containing vesicles.^8^ This involved changes in retrograde signalling via the cannabinoid receptor‐1. Follow‐up studies with conditional deletion of GRs in the PVN (and supraoptic nucleus) revealed the involvement of GRs in these rapid corticosteroid actions.^9^ These rapid GR‐dependent actions in the PVN are important for ACTH and corticosterone responses to acute, but not chronic stress.^10^

In contrast to the PVN, corticosterone was found to rapidly and reversibly *increase* the release probability of glutamate in CA1 hippocampal neurons, with concentrations that are relevant for the stress response, that is around 5‐10 nM.^11^ These rapid non‐genomic effects in the hippocampus were found to be mediated by MR rather than GR; in view of the effective concentration range (which is close to the Kd of the GR but much higher than the Kd of the MR), this was somewhat surprising. In principal neurons of the basolateral amygdala (BLA), MR activation also raised the glutamate release probability within ~10 minutes, but in contrast to the hippocampus, these effects lasted for several hours.^12^ The lasting character in itself depends on translation and involves the GR. Interestingly, the rapid‐onset yet long‐lasting changes in BLA glutamate transmission were accompanied by a change in the response to corticosterone, such that a pulse of corticosterone delivered >1 hour after the first (or after stress) caused a reduction in glutamate release probability; this reduction is caused by a similar mechanism as described for neurons of the PVN, via retrograde endocannabinoid signalling. The phenomenon of a flip in response to corticosterone was dubbed “corticosterone metaplasticity,” indicating that the BLA response to corticosterone depends on the recent stress history of the animal. To what extent other parts of the brain also show rapid responses to corticosterone in addition to the slow gene‐mediated actions is still largely unexplored.

**Figure 2:**
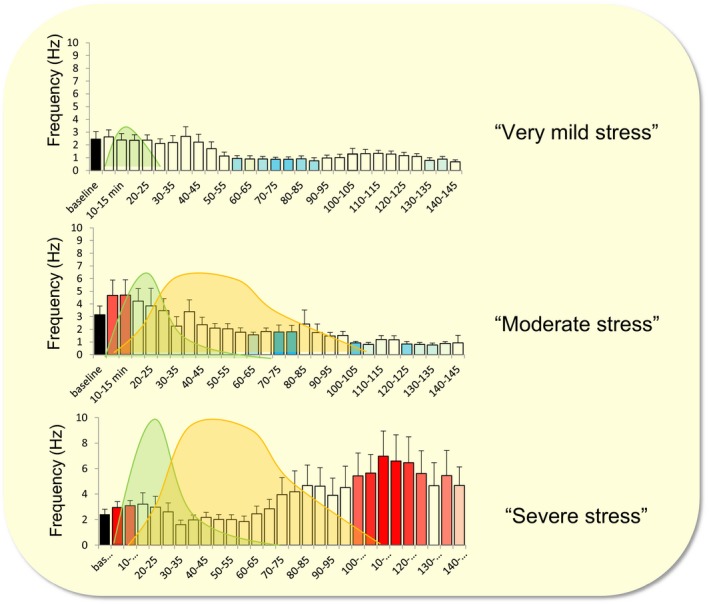
Cellular responses of basolateral amygdala neurons to waves of stress hormones. Basolerateral amygdala cells in vitro were exposed to waves of first isoproterenol (green) and next corticosterone (orange), mimicking the natural variations measured with microdialysis. The top panel shows a brief wave of 0.3 μM isoproterenol (mimicking very mild stress), the middle panel waves of 1 μM isoproterenol followed by 30 nM corticosterone (mimicking moderate stress); and the lower panel the application of 3 μM isoproterenol followed by 100 nM corticosterone (severe stress). The graphs show the averaged (+SEM) frequency of miniature excitatory post‐synaptic currents in time. The intensity of the bar's colour corresponds to the significance of the effect; red bars indicate excitatory responses, blue bars inhibitory responses. The difference between very mild and moderate stress is characterized by the appearance of a brief excitatory response, whereas the shift from moderate to severe stress is associated with the appearance of a delayed excitatory effect. Based on reference 12

A third principle that emerged from cellular studies is the fact that hormones and transmitters released after stress act in concert. They all act in different yet overlapping domains in place and time, depending on the type and severity of the stressor.^13^ The relevance of the overlap is nicely illustrated by the response of BLA neurons to consecutive waves of isoproterenol (acting specifically on the β‐adrenoreceptor which was shown to be highly relevant for behavioural responses to stress) and corticosterone (Figure 2), as occurs after stress.^14^ In this study, the focus was on the frequency of miniature excitatory post‐synaptic currents (mEPSCs), each of which represents the post‐synaptic response to the release of a single glutamate‐containing synaptic vesicle. A very low‐dose brief wave of isoproterenol, mimicking conditions of mild arousal, did not evoke any rapid effects, yet significantly suppressed BLA mEPSC frequency >1 hour later. A sequence of waves of first isoproterenol and then corticosterone, both at moderately high concentrations, as might occur after moderate stress, did quickly raise mEPSC frequency; however, >1 hour after onset of the waves, the mEPSC frequency was changed in a comparable manner as seen with the low isoproterenol dose. The picture was completely different when isoproterenol and corticosterone were consecutively administered at very high concentrations, as might occur with severe stress. Here, a rapid increase in mEPSC frequency became sustained, that is mEPSC frequency remained high for several hours. The results illustrate that waves of these two important stress mediators under conditions that may occur with moderate stress restrain amygdala excitability but that conditions that are relevant for severe stressors are no longer able to do so. Potentially, the latter provides a very long window for encoding of severe stressors which likely involve the amygdala.

All in all, the brain is exposed after stress to waves of various stress hormones which cause a mosaic of regionally different, time‐dependent and interactive changes in cellular physiology.

## 3 FROM CELLULAR PHYSIOLOGY TO BEHAVIOURAL RESPONSES

Ideally, one would like to integrate all of this information at the cellular level, to predict the consequences at the level of circuits and even behaviour. However, the current state of our knowledge does not allow such an integrative approach yet. The level of complexity and many “unknowns” simply do not lead to a reliable model. To nevertheless make an attempt, we tested the hypothesis that stress alters behaviour in a regionally differentiated and time‐dependent manner.

We experimentally approached this in two ways. First, we singled out one hormone by administering hydrocortisone in humans or corticosterone in rodents and tested cognitive performance of thus treated subjects directly after the peak of the corticosteroids—to address rapid non‐genomic actions—or 90‐240 minutes later, to study genomic actions. The second approach was to stress individuals and expose them to cognitive tasks during the rapid and late windows in time. The latter has the advantage of being physiologically more relevant but is more difficult to interpret, given the myriad of hormones and transmitters being released after stress. In some experiments, we made use of stress in combination with corticosteroid receptor antagonists, to specifically investigate the role of each of these receptors in the two time‐domains.

**Figure 3:**
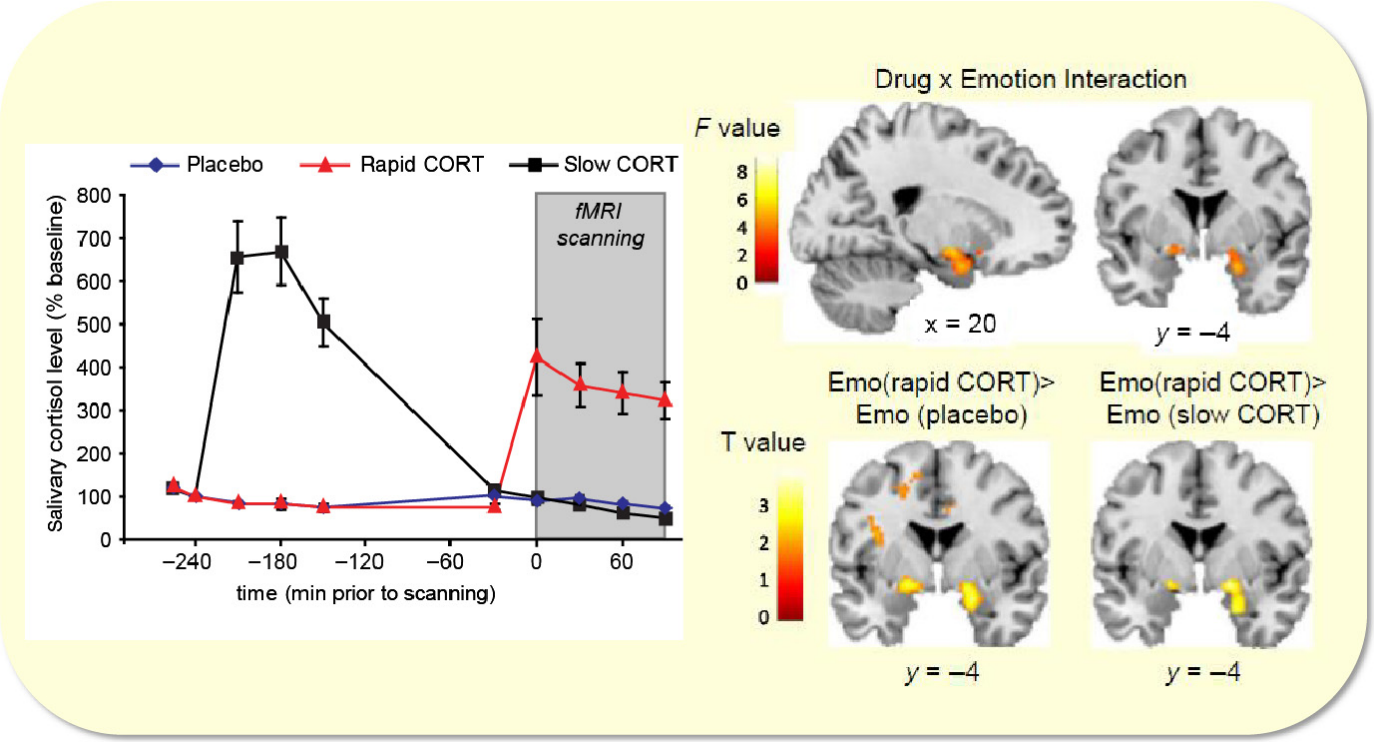
Effect of hydrocortisone on emotional interference. (Left) Participants received two capsules (drug1 and drug2) containing either 10 mg of hydrocortisone (CORT) or placebo at different time‐points before the emotional distraction task. One group received hydrocortisone 4 hours before engagement in the task (black line), another 30 minutes prior to the task (red line). Hydrocortisone intake significantly elevated salivary cortisol levels in both hydrocortisone administration groups compared to the placebo‐placebo control group (blue line). (Right, top) Hydrocortisone administration induced trend of a corticosteroid × emotion interaction in the amygdala. (Right bottom) This interaction appeared to be driven by a significant effect of emotion in the amygdala due to the rapid effects of corticosteroids, suggesting insufficient suppression of emotional interference in this group. The amygdala in the placebo and slow corticosteroid group did not distinguish between the processing of aversive vs neutral words. Adapted from reference 13

Figure 3 shows a typical examples of such experiments in human subjects.^15^ One group (the “slow” effect group) ingested hydrocortisone 4 hours before the task. As shown, hormone levels in this group had normalized at the time of testing, so that presumably, they were too low to induce rapid non‐genomic actions while genomic effects still persisted. Another group received hydrocortisone 30 minutes prior to testing, resulting in sufficiently high levels to induce non‐genomic effects at a moment where genomic actions had not yet developed (the “rapid” effect group). The third group received placebo at both time‐points. In this particular example, subjects were exposed to an emotional Stroop task which tests selective attention to neutral or emotional words. It appeared that the rapid group made fewer correct responses to the aversive compared to neutral words than the placebo or slow group. Concurrently obtained fMRI results demonstrated a drug × emotion interaction in the amygdala. Secondary analysis in the amygdala revealed that for emotional words, the amygdala activation observed in the rapid group was significantly stronger than in the control or slow groups. This trade‐off between emotional/vigilance and executive control circuits was also apparent from a score of other studies, especially when the delay between the peak of cortisol and the moment of testing was taken into account.^16^ The overall observation is that briefly after a peak of cortisol (compared to control conditions), emotional circuits/behaviour is increased while executive control is suppressed; by contrast, several hours after the cortisol peak, the reverse is seen. The crossover is exactly after 1 hour, when rapid effects have disappeared and genomic actions start to evolve.

**Figure 4:**
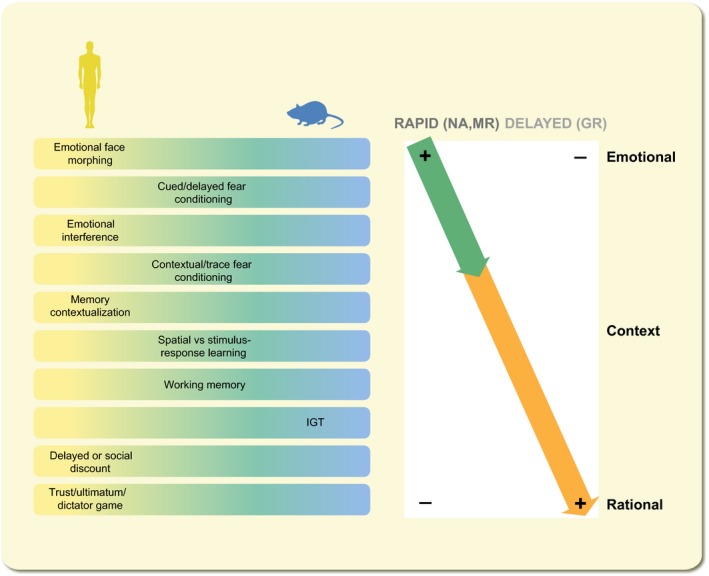
Summary of behavioural observations in rodents and human subjects directly after stress/corticosteroid administration (rapid) and >1 hour after stress/corticosteroid administration (delayed). The tests are arranged from those involving primarily amygdalar/striatal circuits (top), through hippocampal circuits (middle) to prefrontal circuits (bottom). Directly after stress monoamines and corticosteroids acting primarily via MR promote emotional processing, at the cost of higher cognitive functions such as contextual memory formation or reward‐based decision‐making. At a longer interval (>1 hour after stress or corticosteroid administration), the reverse is seen. IGT = Iowa Gambling Task

Other behavioural studies complement this picture. For instance, (social) discounting tasks revealed that directly after a peak in cortisol individuals are more focused on immediate reward and are more generous towards close ones, whereas this is not seen >1 hour later.^17^ These effects were particularly clear when subjects received hydrocortisone alone, rather than being exposed to stress or a mixture of stress hormones, leaving open the possibility that not all stress hormones work in the same direction.^18^ Of note, an increased focus on self does not necessarily mean more selfish behaviour, but merely a sharper distinction between whom to offer costly help and whom not. Also in the realm of contextual memory, cortisol was shown to have time‐dependent effects. Thus, directly after a peak of cortisol, contextual memory and contextualization were found to be decreased, whereas more habitual forms of learning were enhanced.^19^, ^20^ This was associated with an MR‐dependent switch from hippocampal to striatal activity.^21^, ^22^ When subjects were tested several hours later, contextualization was improved in those who received hydrocortisone compared to placebo.^19^ Overall, this leads to the notion (Figure 4) that shortly after a peak in cortisol, individuals are more focused on the “now,” the “self,” on emotional content; and select simple yet inflexible (spatial) solutions. This makes sense from an adaptive point of view because individuals need to be able to act quickly and in the best interest of themselves and their close ones when danger is imminent. Data so far support that this involves rapid actions through MR, in concert with other stress mediators such as monoamines. Interestingly, >1 hour after the peak in cortisol, stressed or hydrocortisone‐treated subjects (compared to controls) show improved executive control and make better use of the context. Given the 1‐hour delay, it seems likely that these actions involve genomic GR‐dependent signalling, but this has not been studied to date in humans. These delayed actions represent an essential secondary phase of the adaptive response because they allow a person to rationalize and contextualize stressful events. One could hypothesize that an imbalance between the two phases leaves the individual vulnerable to major stressors, because the initial response may be too emotional and unrestrained by the control normally exerted by the secondary phase. To what extent such an imbalance indeed is characteristic for those developing psychopathology in the face of major life events remains to be proven.

## 4 BEHAVIOURAL RESPONSES DEPEND ON EARLY LIFE HISTORY

**Figure 5:**
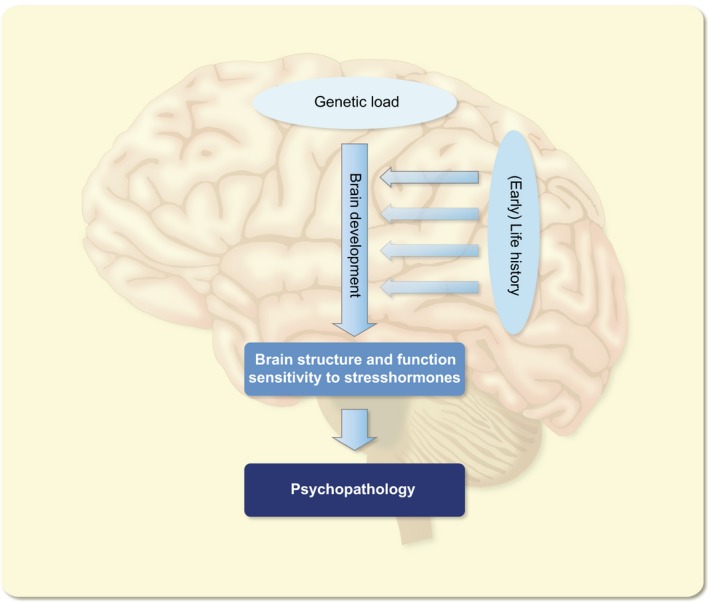
Genetic background in interaction with life events has a strong impact on brain development. This contributes to the wiring and function of the adult brain as well as the functionality of the stress system. In some cases, the brain and stress system have developed such that this may enhance the risk on precipitation of psychopathology, particularly in genetically predisposed individuals

How could such an imbalance between the rapid and delayed phases of the behavioural response to stress develop? Most likely, the system may become gradually unhinged due to an accumulation of life events, particularly in individuals with variations in genes encoding for critical molecules in the stress signalling pathway, associated with aberrant functionality of these molecules, such as has been described for the glucocorticoid receptor or FKBP5.^23^, ^24^, ^25^ Especially, events taking place early in life, when both the stress system and the brain are still developing, are known to have a strong impact.^26^, ^27^, ^28^ This has been studied in detail in rodent models of early life adversity, which have the advantage of control over the genetic background, the (early life) environment and allow detailed investigations of the underlying mechanism (Figure 5). As an illustration of this phenomenon, we will here discuss the effects of early life adversity on contextual memory.

Many studies have shown that perinatal stress impairs the formation of contextual or spatial memory in rodents. Early life adversity models in which this was demonstrated comprise the following: mothers with low levels of licking/grooming and arched‐backed nursing of their litters^29^; mothers exposed to limited bedding and nesting material^30^; and mothers removed for 24 hours from the litter at post‐natal day (P) 3.^31^ Interestingly, impaired contextual/spatial memory formation after early life adversity seems to go hand in hand with enhanced emotional memory or anxiety.^32^ This closely resembles the initial phase of the cognitive stress response sketched above and may point to a permanent imbalance of the (behavioural) stress response. In some of these models, reduced hippocampal expression of MR and GR was demonstrated, sometimes accompanied by elevated corticosterone levels. This is compatible with a bias towards the emotional aspects, at the cost of higher cognitive functions. However, it should be emphasized that the neuroendocrine changes described for the various models are not very consistent and generally only transient in nature. The (i) variability in early life adversity models used, (ii) the generally low sample sizes per experiment and (iii) the many experimental variations in age, brain area and conditions under which corticosterone or receptor measurements were carried out call for a meta‐analytic approach, to come to a more solid conclusion.

The degree to which contextual/spatial memory is impaired depends on many factors. The sex of the animals seems to be important.^32^ A survey of 64 studies in which at least female (and in most cases also male) rodents were tested for cognitive performance after early life adversity, with a total of 212 experimental endpoints, showed that hippocampal learning was impaired in 50% of the studies in male rodents, but only in ~25% of the studies involving female rodents. This may be an age‐dependent phenomenon, because one of the potential underlying substrates—neurogenesis in the dentate gyrus—was found to be strongly suppressed in female rodents shortly after the early life adverse situation, normalizing towards adulthood; whereas, after ELS, neurogenesis was *increased* in male rodents when tested at young age, but suppressed in adulthood.^33^ Consistent inclusion of both male and female offspring in experimental studies is necessary to get the full picture.

**Figure 6:**
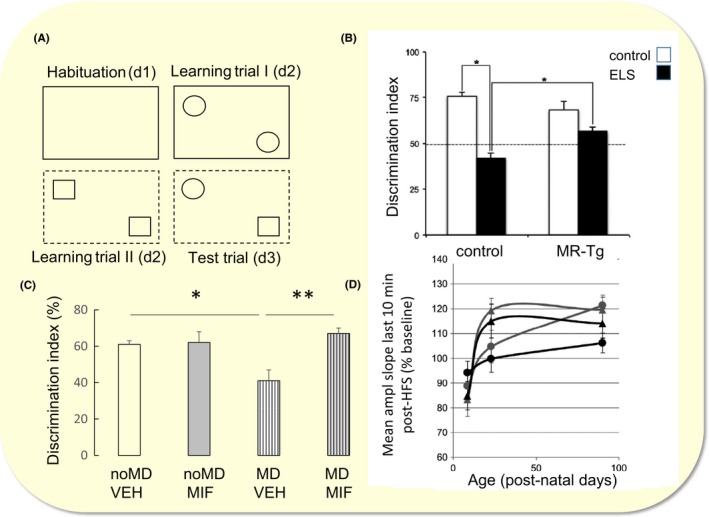
Effect of early life stress on contextual memory, its dependence on genetic background and the possibility to intervene with mifepristone treatment during early puberty. A, Set‐up of the object‐in‐context experiment. Male rats or mice were initially habituated in a context that had no object. Next, during training, the animals were placed in the same context but with two identical objects (learning trial I) and then placed in a novel context with two identical novel objects (learning trial II). Finally, the animals were placed in the latter context but with one object being replaced by an object from the first context (test trial). B, Wild‐type mice exposed to early life adversity showed impaired object‐in‐context learning, as indicated by the significantly reduced discrimination index. This impairment was not observed in mice which had increased MR expression in the brain (MR‐Tg). C, Rats that earlier had been exposed to maternal deprivation showed impaired discrimination between the objects. This was fully restored in animals that had been treated with a GR‐antagonist between days 26 and 28. D, The response to high‐frequency stimulation (HFS) is shown as the mean signal amplitude (P8‐9) or slope (other ages) during the last 10 minutes of the 60 minutes post‐HFS recording (±SEM). Male and female rat data on P8‐9 were pooled after being tested for sex effects on fEPSP baseline characteristics and synaptic plasticity, the other data are based on male rats only. Control animals (circles) showed an increase in response to HFS which continued up into adulthood, while rats exposed to 24‐hours maternal deprivation at P3 (triangles) reached adult levels of long‐term potentiation already at P22‐24. Corticosterone (black lines) compared to vehicle treatment (grey lines) was ineffective, with the exception of male adult rats, where corticosterone impaired the possibility to induce synaptic plasticity. All data expressed as mean ± SEM. Posthoc testing: *P < .05; **P < .01. Based on references (26, 27, 28

Genetic background is a second factor determining the impact of early life adversity on hippocampal function. Genetic variation is limited in inbred mouse strains, but based on the literature, one can specifically investigate the relevance of particular genes, by reducing or elevating the expression level. As an example, when the MR expression level is genetically enhanced—starting around P15—the effects of exposure of the dam to limited bedding/nesting material between P2 and P9 on hippocampal learning in the (adult) offspring were found to be prevented (Figure 6A,B).^34^ Changes in MR relative to GR activation can also be achieved by treating animals with a GR‐antagonist; the timing of this pharmacological approach is very precise (with fewer compensatory effects than genetic modification) but, of course, peripheral administration affects many more organs than just the brain. Using this approach, it was shown that impaired contextual/spatial memory seen in (adult) rats that were exposed to 24‐h maternal deprivation at P3 can be fully prevented by only 3 days of treatment with a GR‐antagonist, between P26 and P28 (Figure 6C).^35^ The impairment was very specific for areas involved in spatial memory and was not seen in areas involved in reward learning. In both studies, glutamate transmission in single hippocampal cells was suppressed by early life adversity per se, but fully restored in the MR‐overexpressing group or animals temporarily treated with the GR‐antagonist. At the field potential level, it was found that 24 hours of maternal deprivation at P3 accelerates maturation of synaptic plasticity, with a critical phase around P22‐24 (Figure 6D).^36^ Possibly, early life adverse conditions target hippocampal glutamate (and most likely GABA) transmission already before puberty which may eventually result in behavioural deficits related to hippocampal impairment.

## 5 CONCLUDING REMARKS

In this review, we have described how acute stress conditions in adults change brain physiology and cognition, in a region‐specific and time‐dependent manner. Rapid non‐genomic actions, involving the MR, may promote the immediate cognitive response to potential threats by amplifying emotional circuit activity and help to focus on the “now” and “self.” Delayed genomic actions most likely via GR are complementary to this immediate response, yet as important to adapt to potential threats in the long run. This later phase helps to put the stressful events in the right perspective, by promoting rationalizing and contextualizing of the event. Although both cellular physiology and cognitive function seem to adhere to the same and compatible principles, it should be appreciated that direct links between cell function and behaviour in relation to acute stress have rarely been attempted in rodents and, to date, are not possible in humans. The principles outlined in this review are therefore based on correlations, similarities in experimental design and pharmacological convergence. However, much more evidence needs to be provided to accept the general applicability of the scheme depicted in Figure 4.

An inference made from this scheme is that an imbalance between the two phases of the stress response may increase the vulnerability to disease, especially in genetically predisposed individuals. The causation of the events, however, has not been proven. One would need to longitudinally sample a large population cohort to prove this. Sequential investigation of the functionality of the stress system, in relation to its impact on cognition, in the face of well‐documented life history (without recall bias) are necessary ingredients to prove that gradual deviations in the effectiveness of one or two phases of the cognitive stress response add to the risk to develop psychopathology. Importantly, most studies so far have been performed at a group level. Yet, individual variations are likely to determine how stress exactly affects the brain and what this means for the vulnerability to disease. Factors such as sex, genetic variation, life history, personality traits and sociocultural environment all contribute to the individual profile.

Finally, it should be noted that many studies to date have been performed in young adult male human subjects or young adult male rodents. The importance of the lifespan is obvious. It is highly relevant *when* adverse life conditions occur relative to (brain) development. There is a great need to determine the potential windows of intervention. And, the ability of the brain to deal with the challenges of life may diminish with age. Clearly, future studies would need to address these aspects as well as to what extent developmental trajectories in rodents are translatable to humans.

## CONFLICT OF INTEREST

The authors report no conflict of interest.
